# P-558. Long-acting Injectable Cabotegravir/Rilpivirine Plus Subcutaneous Lenacapavir Effective in People with HIV with Viremia and Cabotegravir or Rilpivirine Resistance-Associated Mutations

**DOI:** 10.1093/ofid/ofae631.757

**Published:** 2025-01-29

**Authors:** James B Brock, Racolesha Denson, Peyton Herrington, Melissa Hickman, Heather King, Regan McIntosh, Sirna Musa, Courtney Sanders, Holly Walker, Aubri Hickman

**Affiliations:** University of Mississippi Medical Center, Jackson, Mississippi; University of Mississippi Medical Center, Jackson, Mississippi; University of Mississippi Medical Center, Jackson, Mississippi; University of Mississippi Medical Center, Jackson, Mississippi; University of Mississippi Medical Center, Jackson, Mississippi; University of Mississippi Medical Center, Jackson, Mississippi; University of Mississippi Medical Center, Jackson, Mississippi; University of Mississippi Medical Center, Jackson, Mississippi; University of Mississippi Medical Center, Jackson, Mississippi; University of Mississippi Medical Center, Jackson, Mississippi

## Abstract

**Background:**

Long-acting antiretroviral regimens have the potential to achieve virologic suppression in people unable to maintain adherence to daily oral antiretroviral therapy (ART). Long-acting injectable cabotegravir/rilpivirine (LAI CAB/RPV) is currently the only complete long-acting regimen and is recommended by IAS-USA for select patients with viremia. However, resistance-associated mutations (RAMs) among treatment-experienced people with HIV are common, precluding use of LAI CAB/RPV. LAI CAB/RPV plus subcutaneous lenacapavir (LEN) may provide therapeutic options for people with poor long-term adherence and cabotegravir or rilpivirine RAMs.

**Table 1.**
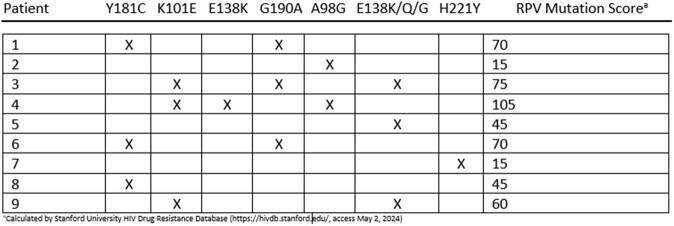
NNRTI Mutations by Patient

**Methods:**

Patients at UMMC’s Adult Special Care Clinic were selected for LAI CAB/RPV plus LEN by a multidisciplinary panel. Criteria for selection included recurrent viremia despite intensive case management strategies, presence of historical or current RAMs to CAB or RPV, and partially- or fully-active cabotegravir based on mutation scores. Patients began therapy with LEN and LAI CAB/RPV on the same day. The prescribed regimen consisted of CAB/RPV 600/900 mg intramuscular at months 0 and 1 followed by every 2 months and LEN 600 mg oral daily x 2 days and 927 mg subcutaneous every six months. No oral lead-in of CAB/RPV was administered, and oral LEN was administered via directly-observed therapy.

**Results:**

Nine patients were treated with LAI CAB/RPV plus LEN at UMMC. Mean baseline viral load was 36 251 copies/mL. All patients had RPV RAMs. One patient had CAB RAMs (N155H, T977A) conferring low-level resistance to CAB. The follow-up period ranged from 24-36 weeks. All patients maintained viral loads < 200 copies/mL during follow-up. Patients with baseline CD4 < 200 cells/µL demonstrated 208% increase in absolute CD4 count. No treatment-emergent resistance has occurred to date.

**Conclusion:**

LAI CAB/RPV plus LEN achieved virologic suppression for up to 36 weeks of follow-up in PWH with incomplete adherence to oral ART and mutations to CAB or RPV. Further follow-up and larger sample size will be needed to determine long-term durability of this combination. Additional combinations of long-acting ART are vitally needed to achieve virologic suppression for all.

**Disclosures:**

**All Authors**: No reported disclosures

